# Effect of very low nicotine content cigarettes on smoking behavior in Chinese smokers with genotype-stratified nicotine metabolism rates

**DOI:** 10.3389/fpsyt.2025.1420650

**Published:** 2025-05-27

**Authors:** Wanwan Ma, Yaning Fu, Huan Chen, Hongjuan Wang, Shulei Han, Yushan Tian, Hongwei Hou, Qingyuan Hu

**Affiliations:** ^1^ Key Laboratory of Tobacco Biological Effects and Biosynthesis, China National Tobacco Quality Supervision and Test Center, Zhengzhou, China; ^2^ Key Laboratory of Tobacco Biological Effects and Biosynthesis, Beijing Life Science Academy, Beijing, China

**Keywords:** very low nicotine content (VLNC) cigarettes, genome-wide SNP genotyping, nicotine metabolism, smoking behavior, Chinese smokers

## Abstract

**Background:**

Smoking is a major public health issue worldwide, particularly in China where it is linked to serious diseases like lung cancer and cardiovascular disease. The genetic basis of nicotine metabolism, which varies significantly between Chinese and Western populations, impacts the effectiveness of interventions aimed at reducing nicotine dependence. Very Low Nicotine Content (VLNC) cigarettes are considered a promising strategy for smoking cessation. However, their effectiveness may vary due to genetic differences, necessitating specific studies on the impact of VLNC cigarettes on Chinese smokers’ nicotine metabolism rates.

**Methods:**

This study utilized genome-wide SNP genotyping to categorize Chinese smokers into slow, medium, and fast nicotine metabolizers based on their genetic profiles. A total of 1,500 healthy volunteers were recruited across seven major Chinese regions and underwent saliva genomic DNA extraction for SNP genotyping. Parts of the participants in Zhengzhou were then provided with study VLNC cigarettes with a nicotine content of approximately 0.7 mg/g and monitored over a four-week period to assess changes in smoking behavior, nicotine exposure and smoking subjective effect.

**Results:**

Transitioning to VLNC cigarettes significantly reduced nicotine dependency in fast and medium metabolizers. Compensatory smoking behaviors, such as increased puff frequency, were particularly noticeable in slow and medium metabolizers. Biomarker analyses confirmed reductions in nicotine intake despite these compensatory behaviors.

**Conclusions:**

VLNC cigarettes effectively reduce nicotine dependency among Chinese smokers, with varying results based on nicotine metabolism rates influenced by genetic differences. The findings underscore the necessity of incorporating genetic profiling into public health strategies for smoking cessation. Further research is required to explore the full scope of compensatory smoking behaviors and to tailor cessation strategies to accommodate genetic variability within the smoker population.

## Introduction

Smoking poses a significant public health challenge globally, with over 300 million affected in China alone. This contributes substantially to the national burden of lung cancer, cardiovascular disease and other chronic illness (1–3). Nicotine, the primary addictive compound in cigarettes, reinforces smoking behavior and underpins tobacco dependence (4, 5). In response to the health risks of tobacco use, regulatory authorities such as the United States Food and Drug Administration (FDA) and the World Health Organization (WHO) have proposed reducing the nicotine content of cigarettes to non-addictive levels to mitigate dependence and facilitate cessation (6, 7). This strategy is supported by addiction research and forms the basis for harm-reduction approaches that aim to lower nicotine exposure without eliminating the smoking ritual.

Very low nicotine content (VLNC) cigarettes, typically containing minimal or non-addictive nicotine levels (around 0.4 mg/g), have emerged as a promising intervention to reduce dependence while maintaining the behavioral and sensory aspects of smoking. Research has demonstrated that switching to VLNC cigarettes significantly reduces nicotine intake, lowers the number of cigarettes smoked, and decreases withdrawal symptoms and craving intensity (8–11). For instance, Donny et al. (2015) reported that individuals using VLNC products exhibited lower consumption and perceived dependence. These findings suggest that VLNC products can help ease the process of smoking cessation (12). However, most existing studies have been conducted in Western populations, and relatively little attention has been given to non-Western smokers. Moreover, while many VLNC studies treat smokers as a homogeneous group, they often overlook the substantial interindividual variability in biological response, particularly differences in nicotine metabolism driven by genetic factors (13, 14). This gap limits the generalizability and effectiveness of VLNC interventions, especially in genetically distinct populations such as Chinese smokers.

Nicotine metabolism plays a critical role in shaping individual smoking patterns and dependence. The rate at which nicotine is metabolized and eliminated from the body varies widely among individuals and is largely regulated by the liver enzyme cytochrome P450 2A6 (CYP2A6) enzymes (15–17). Genetic polymorphisms in CYP2A6 give rise to fast, intermediate, or slow metabolizer phenotypes. Fast metabolizers tend to consume more nicotine to maintain desired levels due to rapid clearance, whereas slow metabolizers retain nicotine longer, which may increase the risk of dependence despite lower intake. These differences influence not only nicotine exposure but also satisfaction, craving, and the likelihood of compensatory smoking behavior when nicotine content is reduced (18–20). Consequently, the success of VLNC interventions may vary significantly depending on a smoker’s metabolic phenotype. Accounting for these differences is essential to fully understand who benefits most from nicotine reduction and to avoid unintended behavioral compensation in certain subgroups.

Importantly, the distribution of CYP2A6 polymorphisms differs markedly between ethnic groups. In particular, several reduced-function or loss-of-function alleles, such as *CYP2A6*4, *7*, and *9*, are significantly more prevalent in East Asian populations compared to Caucasians. For instance, the minor allele frequency of *CYP2A6*4 reaches approximately 25% in Chinese populations, in contrast to 3% in Western populations (21–23). As a result, a substantial proportion of Chinese smokers are likely to be slow or intermediate nicotine metabolizers. These genetic differences suggest that Chinese smokers may exhibit unique behavioral responses to VLNC cigarettes. However, this possibility remains underexplored, and few studies have systematically examined the interaction between nicotine metabolism rate and VLNC response in Chinese individuals. Addressing this gap is essential for understanding the mechanistic basis of VLNC effectiveness and for tailoring tobacco control interventions to population-specific genetic profiles.

This study aims to investigate the influence of nicotine metabolism rate on behavioral responses to VLNC cigarettes in Chinese smokers. Participants were stratified into metabolic subgroups through genome-wide SNP genotyping, and changes in smoking behavior were evaluated using subjective reports and objective biomarkers across different genetic profiles following VLNC cigarette use. This genotype-informed approach offers a more nuanced understanding of the relationship between nicotine metabolism and dependence. The findings have the potential to inform personalized tobacco control strategies, contribute to the development of population-specific cessation policies, and provide a scientific basis for integrating genetic considerations into nicotine reduction initiatives.

## Materials and methods

This study has been approved by Zhengzhou University Hospital’s committee, and all participants gave written informed consent, acknowledging the study’s goals, methods, and risks.

### Study cigarettes

Study VLNC cigarettes with a nicotine content of approximately 0.7 mg/g were developed through a collaboration between Yunnan Tobacco International Co., Ltd. and Jiangsu Xinyuan Tobacco Sheet Co., Ltd. The nicotine reduction process involved sequential extraction with solvents of increasing polarity, specifically petroleum ether, chloroform, acetone, and water, to remove substances from Yunnan-sourced tobacco using an extraction pot (RTN-6.0, Henan, China). The reconstituted tobacco was then dried using hot-air and fluidized drying techniques to obtain the final VLNC filler. The processed tobacco was packed into standard cigarette tubes (84 mm × 24 mm) for subsequent experimental use. The nicotine content of the final product was confirmed by gas chromatography–mass spectrometry (GC-MS), in accordance with internal protocols aligned with CORESTA Recommended Method No. 87 (https://www.coresta.org/sites/default/files/technical_documents/main/CRM_87-April2020).

### Participant

To investigate the impact of VLNC cigarettes on smoking behavior among the Chinese population, CCTV Market Research Co. conducted a nationwide recruitment. The methods employed included telephone interviews, online surveys, and in-person interactions. A total of 1,500 healthy volunteers were successfully recruited from seven major Chinese regions: Shenyang, Beijing, Zhengzhou, Guangzhou, Xi’an, Chengdu, and Shanghai. After obtaining informed consent, these participants underwent questionnaire surveys and genotype analysis. Salivary genomic DNA was extracted from all participants for genetic typing. Based on their genetic types for nicotine metabolism, they were stratified into three groups: slow metabolizers, medium metabolizers, and fast metabolizers.

The recruitment and selection processes adhered to the ethical standards set by the International Medical Organization (CIOMC) and the World Health Organization (WHO). Participant inclusion and exclusion criteria were based on their responses, smoking status, and health conditions. The exclusion criteria included being under 18 or over 60 years of age, participation in other clinical trials within the last 30 days, smoking fewer than five cigarettes daily or using other nicotine products in the past year, plans to quit smoking within the next eight weeks, having severe health issues, recent medication use, or alcohol or substance dependency. From the initial cohort, 600 participants from Zhengzhou were selected for further involvement in the VLNC cigarette trial, 348 met the eligibility criteria and were enrolled in the study. Ultimately, 245 participants successfully completed the entire protocol.

### Genome-wide single nucleotide polymorphism genotyping

This study conducted a feasibility analysis on using saliva samples for genomic DNA extraction and genetic typing. After evaluating various saliva collection and DNA extraction kits, two were selected for preliminary testing: BGI Genomics’ ‘Saliva DNA Collection Kit’ (1000011537) combined with ‘MGIEasy Genomic DNA Extraction Kit (Magnetic Bead Method)’ (1000010524), and Beijing Junno De Biological Technology Co., Ltd.’s ‘Saliva DNA Collection, Preservation, Transportation, and Extraction Kit (Solution Type)’ (2528B). Six saliva samples underwent preliminary testing, and DNA quality and concentration were assessed using the Nanodrop 2000 ultramicro UV-Vis spectrophotometer. The BGI Genomics kits demonstrated superior extraction quality, meeting the requirements for further genetic analysis.

Based on the feasibility results, saliva DNA was extracted in the main study using the Oragene•DNA collection system (DNA Genotek). DNA quality was further verified by spectrophotometry and agarose gel electrophoresis. Genome-wide SNP genotyping was conducted using the Illumina Global Screening Array. Quality control steps included excluding SNPs with call rates below 98% and removing samples with poor call rates or inconsistent metadata. This process ensured reliable and high-quality genotypic data for subsequent metabolic grouping and association analyses.

### Study design and procedure

The study utilized genome-wide SNP genotyping to analyze SNP sites related to nicotine metabolism among 1500 participants. From these results, the distribution of nicotine metabolism gene subtypes within the Chinese population was determined. Consequently, metabolic types and corresponding genetic polymorphisms were identified, leading to the development of a Genotype-based Nicotine Metabolism Grouping Model. Based on this model, a genotype-based nicotine metabolism grouping model was developed to classify individuals into slow, medium, and fast metabolizers. Of the 1,500 genotyped participants, 600 individuals from Zhengzhou were shortlisted for feasibility of in-person follow-up. Following inclusion and exclusion screening, a total of 245 participants completed the VLNC cigarette intervention trial. The distribution of nicotine metabolism genotypes and baseline behavioral characteristics were comparable between the initial screened cohort and the final sample, supporting the representativeness of the intervention cohort.

After being grouped, participants were enrolled in a four-week trial where they continued smoking their regular cigarette brands for the first two weeks to establish a baseline. For the following two weeks, they were provided with study VLNC cigarettes to investigate the impact on smoking behavior in Chinese smokers with genotype-stratified nicotine metabolism rates. Weekly clinic visits were scheduled to assess tobacco usage and other substance use. During these visits, levels of exhaled carbon monoxide (CO) and carboxyhemoglobin (COHb) were systematically recorded with a portable PICO+™ Smokerlyzer^®^ device (Bedfont Scientific Ltd., UK). Urinary NNAL, nicotine, and its metabolites, cotinine and 3-OH-cotinine, were measured using Liquid Chromatography with Tandem Mass Spectrometry (LC-MS/MS), as previously described (24). A 0.3 mL urine sample was processed by protein precipitation and filtration, and 5 µL of the prepared extract was injected for analysis. Behavioral changes, including modifications in cigarette consumption, puff intervals, and puff duration, were closely monitored. Participants’ subjective experiences were evaluated through the Questionnaire of Smoking Urges (QSU), which assessed various dimensions of subjective effects, such as relief of withdrawal symptoms, intention to smoke, total score of smoking desire, and expectations of positive effects. To accommodate any potential increases in cigarette usage, the quantity of provided cigarettes was doubled. Participants received these cigarettes free of charge and were compensated for their participation, including attending clinic visits and completing questionnaires.

### Statistical analyses

To assess the impact of switching to VLNC cigarettes on indicators like smoking behavior, subjective effects, and biomarkers, the study computed the mean ± standard deviation for each indicator pre- and post-transition. Clear visual representations of the data were created using OriginPro 9.0. Given that the transition involved a single variable (switching to VLNC), a one-way ANOVA was applied to explore differences in subjective effects, smoking behaviors and exposure among various genotypic groups pre- and post-VLNC transition. These analyses helped identify significant changes attributable to the VLNC switch and the interaction between the switch and genotype.

## Results

### Nicotine metabolism typing model based on SNPs

The genetic analysis of 1,500 individuals, detailed in **Table 1**, elucidates the distribution of nicotine metabolism gene subtypes within the Chinese population. The determination of metabolic types and corresponding genetic polymorphisms was informed by the roles of CYP2A6 and CYP2B6 in nicotine metabolism, as well as by documented activities of these subtypes (20, 32, 33). CYP2A6*1, associated with fast nicotine metabolism, is prevalent in 66.20% of the population. Subtypes CYP2A6*4 and CYP2A6*9, indicative of slow metabolism, are found in 11.70% and 22.10%, respectively. For fast metabolism, CYP2B6*1 and CYP2B6*2 are common in 44.87% and 6.49% of the population. Medium metabolism rates are linked to 13.82% with CYP2B6*4 and 34.82% with CYP2B6*6, involving mutations rs2279343 and a combination of rs3745274 and rs2279343. Classifications are grounded in genetic markers, with CYP2A6*4 deletions and rs28399433 mutations signaling slow metabolism, while a single rs28399433 mutation suggests medium metabolism. The presence of two copies of rs2279343 or one copy alongside rs3745274 mutations also indicates medium metabolism.

**Table 1:** SNPs associated with nicotine metabolism and typing results in the Chinese population.

Gene	Gene subtype	Types of gene polymorphism	Proportion	Metabolic type	Ref
CYP2A6 **(70-80%)**	CYP2A6*1	wild-type allele	66.20%	Fast	(15, 16, 25–27)
	CYP2A6*2		0%	/	
	CYP2A6*3		0%	/	
	CYP2A6*4	CYP2A6 deleted	11.70%	slow	
	CYP2A6*5		0%	/	
	CYP2A6*6		0%	/	
	CYP2A6*7		0%	/	
	CYP2A6*8		0%	/	
	CYP2A6*9	rs28399433	22.10%	slow	
	CYP2A6*10		0%	/	
CYP2B6 **(10-20%)**	CYP2B6*1	wild-type allele	44.87%	Fast	(28–31)
	CYP2B6*2	rs8192709	6.49%	Fast	
	CYP2B6*3		0%	/	
	CYP2B6*4	rs2279343	13.82%	medium	
	CYP2B6*5		0%	/	
	CYP2B6*6	rs3745274, rs2279343	34.82%	medium	
	CYP2B6*7		0%	/	
	CYP2B6*8		0%	/	
	CYP2B6*9		0%	/	
	CYP2B6*10		0%	/	

The asterisk (*) names a specific allele or variant of the CYP2A6 or CYP2B6 gene.

### Analysis of representativeness of the study population

**Figure 1:**
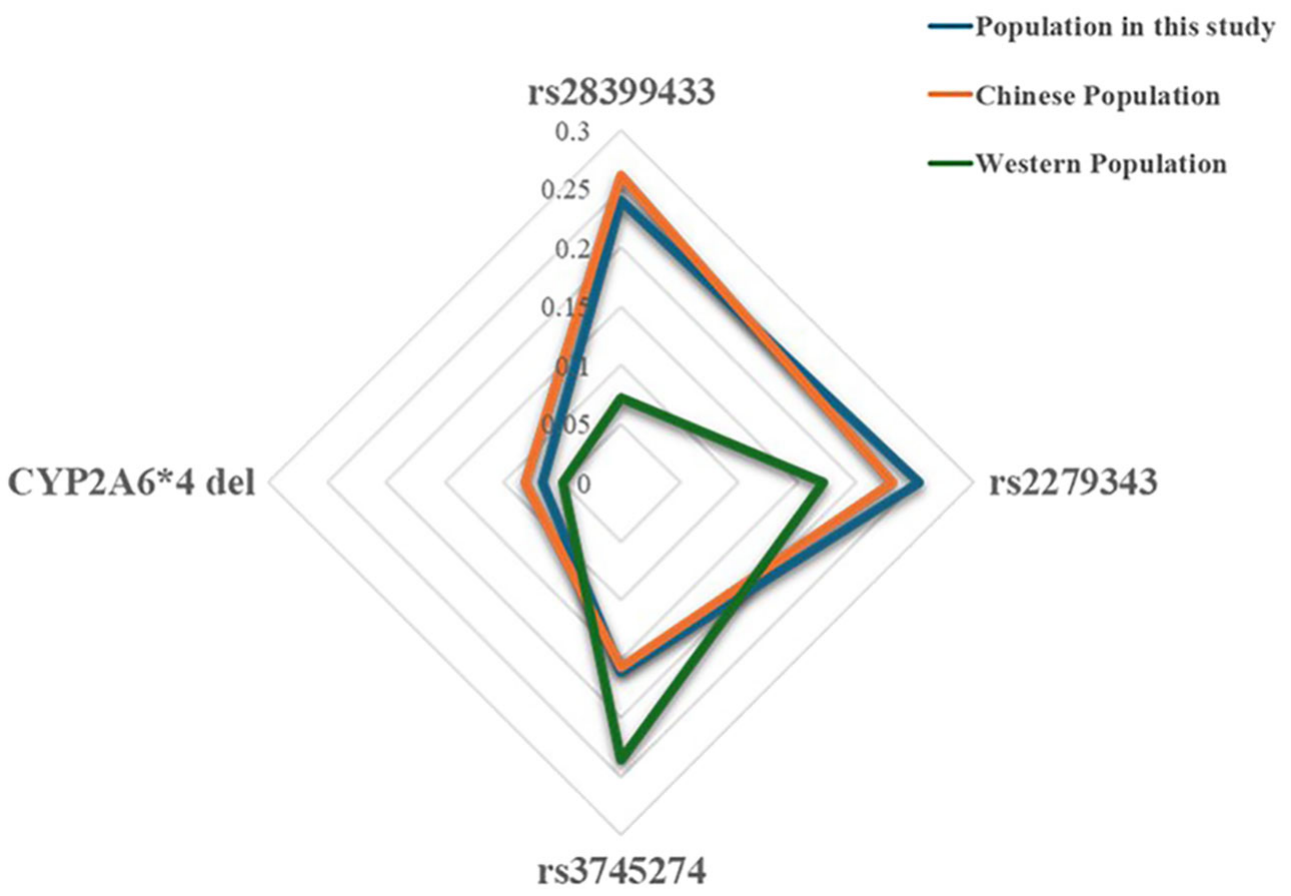
Analysis of MAF of genetic polymorphic loci used in the models among Chinese and Western populations.

The genotype-based nicotine metabolism grouping model utilized in this study is based on four key genetic polymorphisms: CYP2A6*4 del (a deletion mutation), rs28399433, rs2279343, and rs3745274. To analyze global allele distributions, the study utilized resources from the International Genome Sample Resource (IGSR), including the 1000 Genomes Project, and data from the National Center for Biotechnology Information (NCBI). **Figure 1** demonstrates the representativeness of the subjects, revealing that the MAF values for these loci in the Chinese cohort are in line with national data but distinct from those in Western populations. This indicates that the selected subjects accurately represent Chinese genetic diversity and may respond differently to VLNC cigarettes than Western populations.

### Validation of the genotype-based nicotine metabolism grouping model

**Figure 2:**
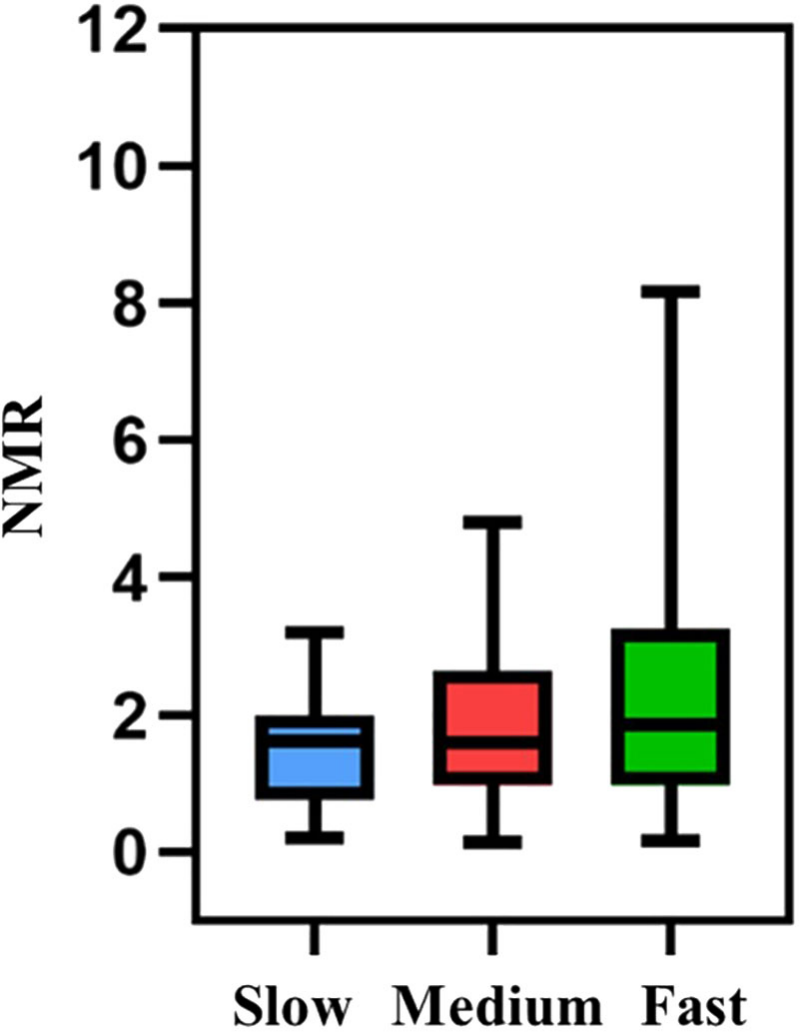
NMR scores in different genotype-based nicotine metabolism groups.

In this study, the 245 eligible participants from Zhengzhou were categorized into fast, medium, and slow metabolism groups based on a nicotine metabolism typing model. Validation was conducted using the nicotine metabolite ratio (NMR) (34), specifically the 3-OH-cotinine to Cotinine ratio. The NMR findings were consistent with the genotypic trends, confirming the model’s validity (**Figure 2**).

### Based characteristics of participants

From 600 individuals initially screened, participants were categorized into slow, medium, and fast nicotine metabolism groups based on genotype. The distribution was as follows: 106 slow metabolizers (17.7%), 284 medium metabolizers (47.3%), and 210 fast metabolizers (35.0%). Ultimately, 245 participants completed the study, comprising 38 slow metabolizers (15.5%), 119 medium (48.6%), and 88 fast (35.9%). The completion rates closely aligned with their initial representation, indicating a balanced dropout rate across all groups.

As shown in **Table 2**, this study examined various smoking behaviors and exposure biomarkers across these groups and found no significant differences. The duration of a single puff, the interval between puffs, and the volume of smoke inhaled per puff were consistently similar across all groups, with p-values of 0.6845, 0.6398, and 0.3291 respectively. Similarly, baseline biomarkers such as CO, COHb, and various nicotine metabolites including cotinine/creatinine, 3-OH-cotinine/creatinine, nicotine/creatinine, and NNAL/creatinine showed no significant differences, with p-values ranging from 0.2205 to 0.8007. Moreover, the QSU assessed the subjective experience of smoking and found similar patterns among the groups. Measures such as relief of withdrawal symptoms, intention to smoke, total score of smoking desire, and expectations of positive effects from smoking all showed no significant variations, with p-values from 0.2112 to 0.9653. These results suggest that at baseline, there are no notable behavioral or biological differences among slow, medium, and fast metabolizers in how they smoke and respond to nicotine exposure, indicating that any subsequent changes observed during the study are likely due to the intervention (switching to VLNC cigarettes) rather than pre-existing differences.

**Table 2:** Participant socio-demographics, smoking behavior characteristics, and biomarkers of exposure at baseline by moderator subgroups, Mean ± SD.

Indicator	Genotype-based Nicotine Metabolism Groups			*p*
	Slow	Medium	Fast	
**Total inclusion**	106(17.7%)	284(47.3%)	210(35.0%)	/
**Completed participants**	38(15.5%)	119(48.6%)	88(35.9%)	/
**Age (years)**	30.32 ± 10.4	36.65 ± 11.17	36.94 ± 11.04	0.0044(**)
**Years smoked**	12.19 ± 10.35	18.45 ± 10.45	18.14 ± 9.7	0.0038(**)
**Single puff duration (s)**	4.04 ± 1.14	3.89 ± 1.34	3.82 ± 1.19	0.6845
**Puff interval (s)**	24.34 ± 12.71	26.36 ± 11.1	25.62 ± 11.15	0.6398
**Single puff inhalation volume (mL)**	75.15 ± 32.91	70.43 ± 27.72	66.26 ± 29.49	0.3291
**CO(ppm)**	15.08 ± 5.26	16.14 ± 5.5	15.28 ± 5.81	0.4353
**COHb(%)**	3.04 ± 0.84	3.21 ± 0.88	3.1 ± 0.94	0.5069
**Cotinine/Creatinine (ng/mg)**	5.41 ± 4.26	5.49 ± 5.43	6.74 ± 5.45	0.2205
**3-OH-cotinine/Creatinine(ng/mg)**	12.14 ± 12.2	10.4 ± 9.98	10.72 ± 10.13	0.6850
**Nic/Creatinine (ng/mg)**	10.02 ± 8.93	7.8 ± 7.84	8.58 ± 7.88	0.3786
**NNAL/Creatinine (ng/mg)**	3.62 ± 2.37	3.78 ± 3.28	3.98 ± 2.63	0.8007
**QSU-Relief of withdrawal symptoms**	13.05 ± 4.81	13.21 ± 4.05	13.26 ± 3.47	0.9653
**QSU-Intention to smoke**	10.58 ± 4.94	11.76 ± 4.86	11.8 ± 4.42	0.3566
**QSU-Total score of smoking desire**	9.16 ± 4.44	10.74 ± 5.83	11.06 ± 5.84	0.2112
**QSU-Expectations of positive effects**	11.66 ± 4.44	11.97 ± 4.84	12.15 ± 4.73	0.8675

### Acceptance of the genotype-based nicotine metabolism groups

**Figure 3:**
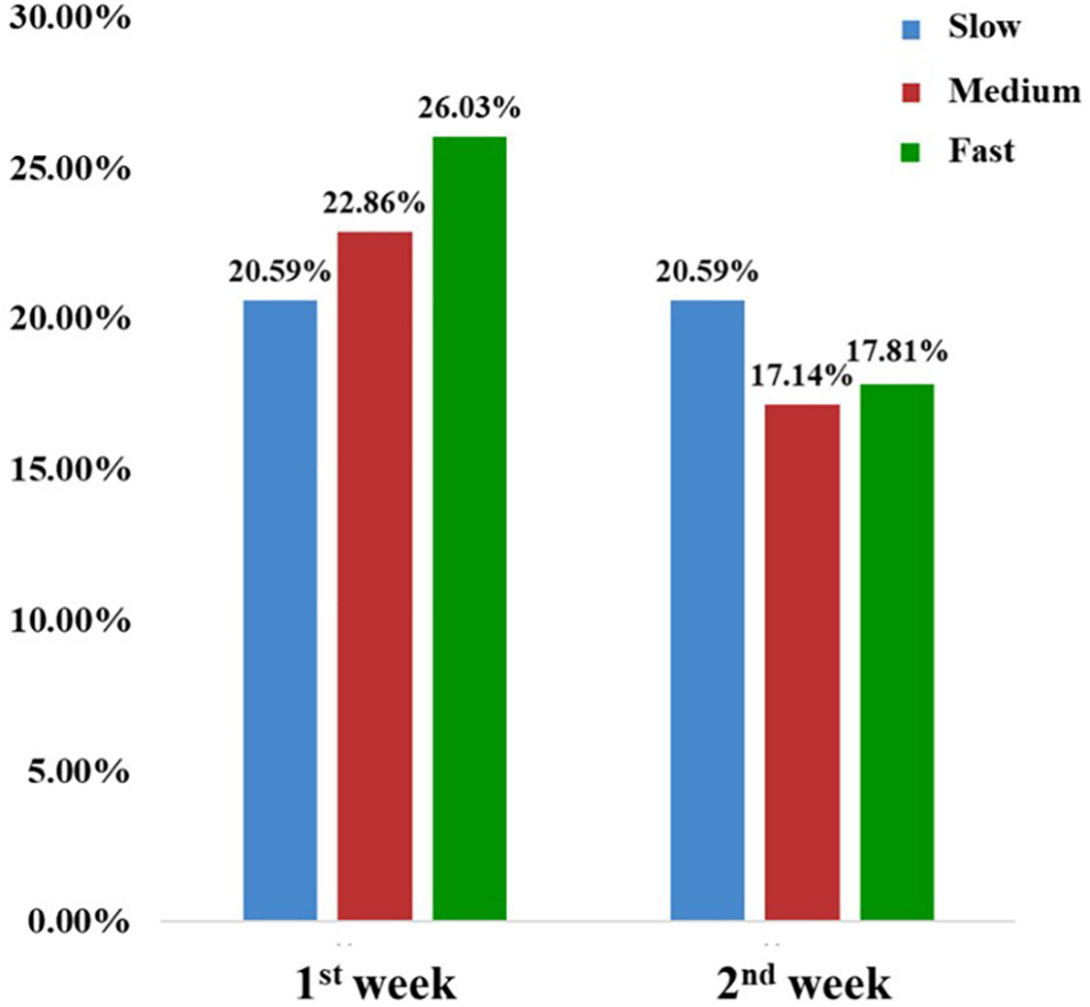
Compliance rates among participants with different nicotine metabolism types.

Acceptance of 0.7 mg/g low-nicotine cigarettes varied among different genotype-based nicotine metabolism groups over two weeks, as depicted in **Figure 3**. Initially, compliance rates were similar across slow, medium, and fast groups, with the medium group showing the highest acceptance at 26.03%. By the second week, compliance declined in all groups. The fast metabolism group’s rate fell to 17.81%, a significant reduction from the first week, while the slow group showed improved adaptation with a compliance rate of 20.59%. These results highlight the impact of genotype on acceptance and compliance with reduced nicotine cigarettes, particularly the marked change in the fast metabolism group.

### Smoking behavior and exposure

**Figure 4:**
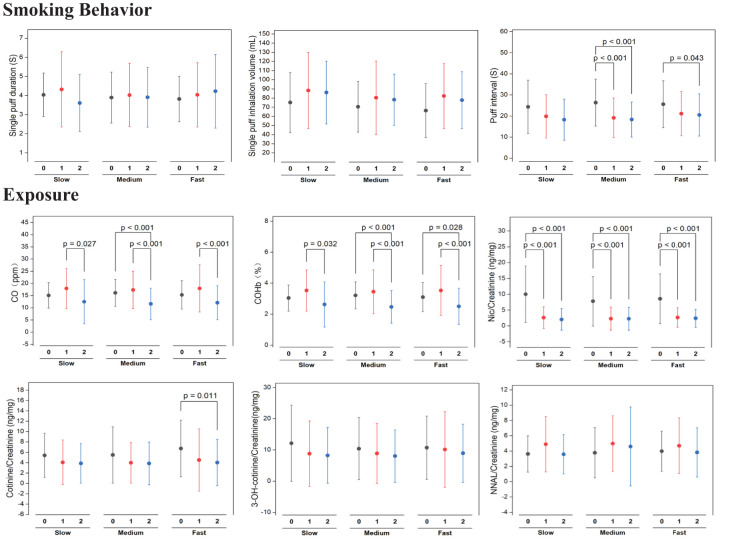
Statistical significance of pre (week 0) switching to study VLNCs and after (1^st^ week and 2^nd^ week) on smoking behavior and exposure of smoking across genotypic based nicotine metabolism rate groups.

**Smoking Behavior:** The study evaluated the smoking behaviors of slow, medium, and fast nicotine metabolizer groups after transitioning to cigarettes with VLNC cigarettes. As shown in **Figure 4**, measurements of puff duration, inhalation volume, and puff intervals were recorded over two weeks. While puff duration and inhalation volume displayed variability, neither exhibited significant changes across all groups, suggesting these aspects of smoking behavior remained stable post-switch to VLNC cigarettes. Conversely, puff intervals showed a statistically significant decrease in both slow and medium metabolizers (p < 0.001), indicating an increase in puff frequency from the onset and maintained through the second week. The fast metabolizers presented a significant decrease in puff intervals by the second week (p = 0.043), hinting at a delayed but notable behavioral adjustment.

**Biomarkers of exposure:** As shown in **Figure 4**, All nicotine metabolism groups (slow, medium, and fast) exhibited a significant increase in exhaled CO levels after switching to VLNC cigarettes, with the levels decreasing by the second week but not returning to baseline. Specifically, slow metabolizers showed a significant rise in the first week (p = 0.027), medium metabolizers saw an initial sharp increase (p < 0.001), and fast metabolizers also had a marked rise (p < 0.001), with all groups maintaining elevated levels compared to the baseline. The percentage of COHb in the blood increased initially across all groups, with a reduction in the second week, yet remaining above baseline for medium (p < 0.001) and fast (p = 0.028) metabolizers. Nicotine levels significantly dropped from baseline to the first week, a decrease sustained into the second week across all groups (p < 0.001). Cotinine levels followed a similar pattern, especially notable in medium metabolizers with a significant decrease (p = 0.011). Levels of 3-OH-cotinine and NNAL, both metabolites of nicotine, showed little change, indicating stable metabolite levels during the study period.

### Subjective effects of smoking

**Figure 5:**
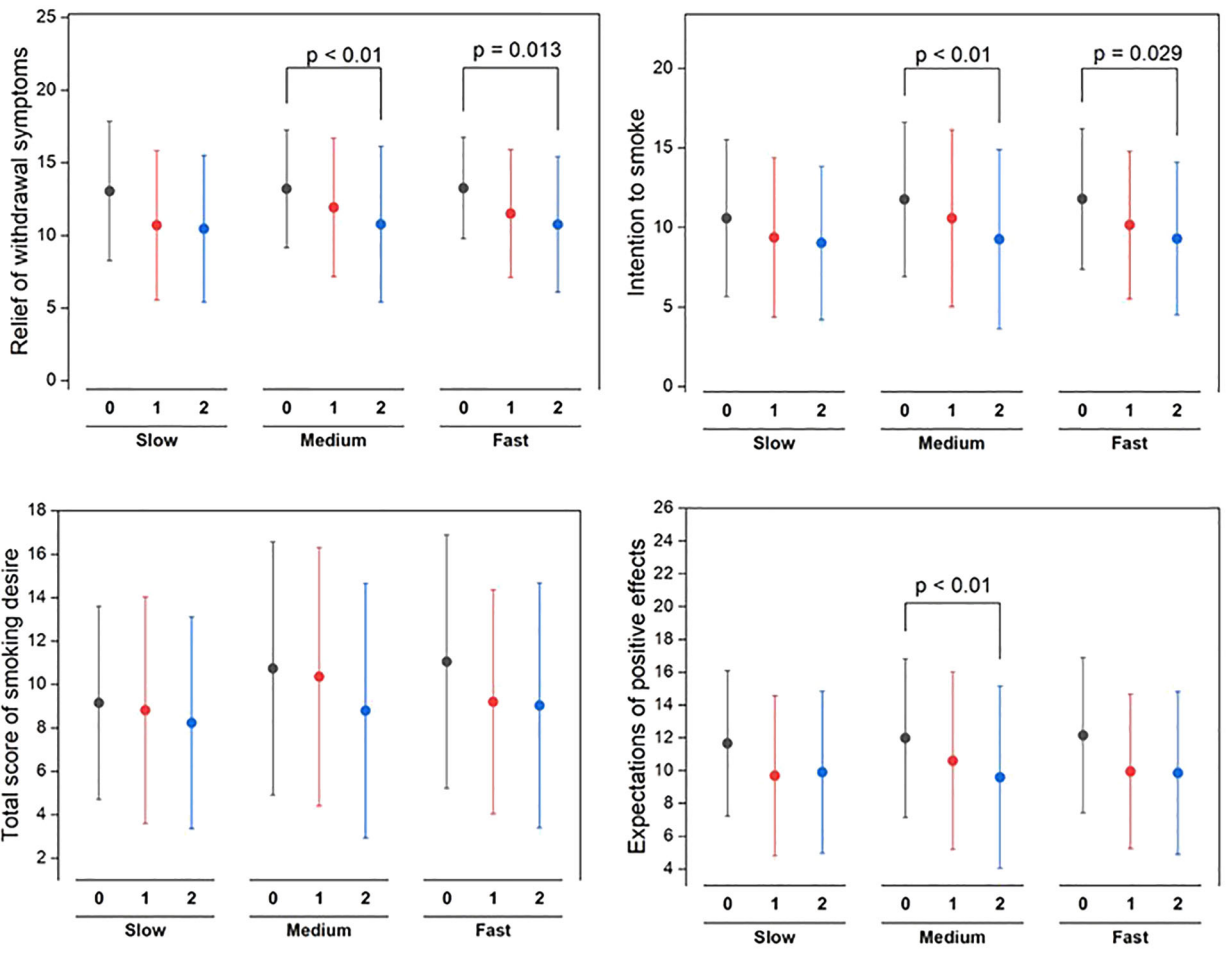
Statistical significance of pre (week 0) switching to study VLNCs and after (1^st^ week and 2^nd^ week) on subjective effects of smoking across different nicotine metabolism rate groups.

**Figure 5** depicts a clear pattern of change in subjective smoking experiences among different nicotine metabolism rate groups upon switching to VLNC cigarettes. Both the medium and fast metabolizer groups experienced significant reductions in withdrawal symptom relief, with p-values of less than 0.01 and 0.013 respectively, indicating less alleviation of symptoms after switching to VLNC cigarettes. Additionally, these groups exhibited notable declines in their intention to smoke, particularly after the first week, with the medium group showing a p-value of less than 0.01 and the fast group at 0.029. Although the total smoking desire score showed variability, there were no significant changes, indicating that smoking desire remained stable across different nicotine levels. The medium group, in particular, reported a significant decrease in their expectations of positive effects from smoking (p < 0.01), suggesting a shift in their perceptions of smoking benefits with VLNC use.

## Discussion

In this study, by identifying specific genetic polymorphisms associated with the CYP2A6 and CYP2B6 enzymes, researchers effectively categorized smokers into fast, medium, and slow metabolizers. These genetic factors play a crucial role in determining the rate of nicotine metabolism, which in turn affects smoking behavior. After switching to VLNC cigarettes, smokers exhibited significant behavioral adaptations, particularly a reduction in nicotine dependency observed in the fast and medium metabolizer groups, consistent with the expected outcomes of using VLNC cigarettes to facilitate smoking cessation.

Despite differences in nicotine metabolism, VLNC use led to observable changes in smoking patterns. Among slow and medium metabolizers, decreased puff intervals indicated an increase in puff frequency, likely reflecting compensatory behavior to maintain nicotine levels. In fast metabolizers, this behavioral adjustment occurred more gradually, with significant changes emerging only in the second week. Importantly, puff duration and inhalation volume remained stable across all groups, suggesting that fundamental smoking mechanics were not altered. Research (35) also indicates that individuals often modify their smoking behavior when smoking VLNC cigarettes to compensate for reduced nicotine intake. Although participants were instructed to use only study-provided VLNC cigarettes, unmonitored tobacco use or variability in psychological readiness to quit may have influenced behavioral outcomes. Furthermore, the compensatory behavior observed in slow metabolizers may be explained by both physiological and psychological mechanisms. Physiologically, delayed nicotine clearance may lead to mismatches between expected and actual reinforcement, prompting more frequent puffing. Psychologically, the reduced reward associated with VLNC cigarettes may trigger elevated craving or habitual responses, particularly in individuals accustomed to higher nicotine delivery. This behavioral compensation reflects the complex interplay between pharmacokinetics and reinforcement mechanisms in shaping smoking behavior under nicotine-reduced conditions. Although the QSU is a validated measure of craving, its self-reported nature introduces potential bias. Future research may consider incorporating qualitative methodologies to better capture individual variability and provide deeper insight into the subjective experience of nicotine reduction. Additionally, *post hoc* analyses did not identify statistically significant differences between metabolism groups (data not shown). This may be explained by high intra-group variability, the short duration of the intervention, and the influence of psychosocial and contextual factors beyond nicotine metabolism. These results emphasize the value of focusing on within-group behavioral adaptations, which presented distinct temporal dynamics despite the absence of inter-group significance.

These findings not only emphasize the importance of individual genetic differences in public health strategies but also provide a scientific basis for future smoking cessation strategies tailored to specific genetic backgrounds. Studies like those conducted by Chen et al. (36) have shown that faster metabolizers may experience more intense withdrawal symptoms, which could affect their smoking cessation success. However, the lack of significant variation in withdrawal symptoms and smoking intention across groups at baseline in this study suggests that the VLNC intervention could be equally effective across different nicotine metabolism rates. The results of this study are consistent with previous studies (37, 38), suggesting that the potential benefits and minimal harms of VLNC cigarettes extend to both slow and normal metabolizers. However, the attenuation of positive reinforcement provided by VLNC may influence how normal metabolizers respond to nicotine reduction.

In the Chinese population, the high prevalence of slow nicotine metabolizers is not only a unique physiological characteristic but also a critical factor that public health interventions need to specifically consider. This genetic predisposition may significantly affect the effectiveness of VLNC cigarettes in the Chinese population. Compared to Western populations, where fast metabolizers are more common, the higher proportion of slow metabolizers in China might impact the smoking cessation effects of VLNC cigarettes. Research (39, 40) indicates that there are significant differences in CYP2A6 gene polymorphism among different ethnic groups, which may explain why there are more slow and medium metabolizers in the Chinese population.

In conclusion, while VLNC cigarettes significantly reduced nicotine dependency across all groups, the most pronounced behavioral adaptations were observed in fast and medium metabolizers. Conversely, slow metabolizers exhibited more compensatory smoking behaviors, such as a marked increase in puff frequency, to maintain nicotine intake levels. This suggests a differential impact of VLNC cigarettes based on genetic nicotine metabolism rates, highlighting the need for personalized approaches in smoking cessation strategies. In addition to biological factors, future research should consider how broader social determinants such as socioeconomic status, cultural norms, and health literacy may influence smokers’ behavioral responses to nicotine reduction. Individuals from lower socioeconomic backgrounds may face greater stress and reduced access to cessation resources, potentially affecting adherence to low-nicotine products. Cultural beliefs may shape receptiveness to behavioral interventions, while varying levels of health literacy can affect the ability to interpret and act on health information. Integrating these contextual variables into nicotine reduction research may enhance the relevance, equity, and overall effectiveness of population-level tobacco control strategies (41, 42).

### Limitations

The study effectively categorized smokers by their genetic profiles and observed significant behavioral adaptations after switching to VLNC cigarettes. However, the intervention period was limited to two weeks of VLNC use, which may not have been sufficient to observe longer-term adaptations or cessation outcomes. Future studies should consider extending the follow-up duration to evaluate sustained behavioral and physiological responses to nicotine reduction. Additionally, compensatory smoking behaviors were not comprehensively captured or controlled. This oversight could include factors such as variations in the depth of inhalations, the number of cigarettes smoked outside the controlled settings of the study, or other compensatory behaviors not measured, all of which could influence nicotine intake and overall satisfaction from smoking. Future studies should aim to address these behaviors more comprehensively. This would not only help in understanding the nuanced impacts of VLNC cigarettes across different genotypes but also in evaluating the real-world effectiveness of reduced nicotine content strategies. Such an approach could provide a deeper insight into the complexities of smoking cessation interventions and their effectiveness across diverse populations.

## Conclusions

This study provides insights into the impact of genetic factors on smoking behavior changes with VLNC cigarettes among Chinese smokers. Using genome-wide SNP genotyping, smokers were categorized into slow, medium, and fast metabolizers. VLNC cigarettes were found to significantly reduce nicotine dependency in fast and medium metabolizers, supporting their potential in smoking cessation. The findings emphasize the need for personalized public health strategies that account for genetic differences in nicotine metabolism. The prevalence of slow metabolizers in China highlights the importance of considering genetic traits in smoking cessation interventions. Further research is necessary to explore compensatory behaviors and the effectiveness of VLNC cigarettes across diverse populations.

## Data Availability

The raw data supporting the conclusions of this article will be made available by the authors, without undue reservation.
